# 

**Published:** 1887-04

**Authors:** 


NINTH INTERNATIONAL MEDICAL CONGRESS.—In reference to demands from various quarters for information as to hotel rates here in Washington, and what arrangements have been made for a reduction of the same, by our committee, in favor of those who will attend the International Medical Congress, I beg to announce the following scale of prices:

| | |
|---|---|
| The Arlington Hotel, from - - - - - | $3 to 3.50 per day. |
| Riggs House, from - - - - - - - - | 3 to 3.50 per day. |
| Willard's Hotel, from - - - - - - | 3 to 3.50 per day. |
| Metropolitan Hotel, - - - - - - - - - - | 3.00 per day. |
| National Hotel, - - - - - - - - - - - | 3.00 per day. |

Other hotels, conducted on European style, will furnish rooms at from $1 to $2 a day. First-class lodging houses will also furnish rooms from $1 to $1.50 a day.

A. Y. P. GARNETT, M. D.,

*Ch'n Com. of Arr. Int. Med. Congress.*

1319 New York Ave., Washington, D. C., March 14, 1887.
—*Journal Am. Med. Association.*

### THE MEDICAL ASSOCIATION OF GEORGIA.

The next annual meeting of the Medical Association of Georgia, to be held in this city, commencing April 20th, promises to be one of the most interesting as well as one of the most important meetings of that body ever yet held. It will be a meeting in which every member of the regular profession in this State will be directly interested. Not alone is the profession of Georgia interested in the proceedings of the forthcoming meeting, but the profession elsewhere—the profession at large. *The Alabama Medical and Surgical Journal*, in reviewing the volume of Transactions for last year, says: "But few Southern States can boast of so many scientific and medical men as Georgia, but it needs better organization, and it is to be hoped that the Medical Association will be instrumental in binding the profession of the entire State in the most effectual manner possible, so that, by a united effort, it may be enabled to influence the enactment of laws which will prohibit the ingress of so many incompetents, and, too, free the State from so many quacks and imposters. Let the requirements be rigid, and then only men of high attainments will apply for admission. Dr. Love, the fraternal delegate to the Alabama Medical Association, spoke in the most complimentary terms of the work of the Alabama Association, and recommended that the Georgia Association pattern after this one. . . . We trust that the Georgia Association will not remain quiet, but will take active steps toward a more perf ect organization."

It is contemplated to take these "active steps" at the meeting in April, and it is earnestly urged upon the members of the regular profession throughout the State to come to that meeting, whether they are or are not members of the Association. If they are not members, they should come in and aid in bringing about "a more perfect organization." Their interest is a common interest. The profession must be upheld *by the profession.* This can be done only by union and concert of action. Now is the time for them to show their hands. The report and recommendation of Dr. Love, alluded to above, met a most cordial reception at the last annual meeting, and will be brought up for the consideration and action of the Association at this. They contemplate an entire re-organization, and, if carried out, will redound to the honor and interest of every member of the profession throughout the State, unite the fraternity in a more solid phalanx, and in the end elevate the standard of that profession. We cannot too strongly urge upon all interested in this elevation to be present and give their aid in the more thorough organization. Aside from the questions presented above, the usual programme of the annual meetings of the Association, which have increased in interest from year to year, will be carried out.

### OUR ADVERTISERS.

SEE new advertisement of Florida R. & N. Co.

SEND to Isaac Phillips, Atlanta, Ga., for new price-list of surgical instruments just out.

CHAS. O. TYNER, Atlanta, Ga., invites the profession to call and inspect his stock of surgical instruments and appliances.

CODMAN & SHURTLEFF, BOSTON.—See advertisement of this well-known firm and write for anything you may need in their line.

FELLOWS' SYRUP HYPOPHOSPHITES.—There is no preparation of the kind so well known or so extensively used in this country as Fellows' syrup hypophosphites. It gives general satisfaction.

WM. R. WARNER & CO., PHILADELPHIA.—No firm stands better with the profession of this country than this one. Their goods are all first-class. Their ingluvin is superior to any in the market.

TONGALINE.—Dr. C. J. Gessey, Germantown, Ill., writes: It affords me great pleasure to inform you that I have used tongaline for rheumatic gout and neuralgic headache, and it has afforded speedy relief in several instances.

GALVANO-FARADIC MANUFACTURING CO., NEW YORK.—This company has an attractive advertisement in this number of THE JOURNAL, to which we desire to call the attention of our readers. These batteries are extensively used and give satisfaction.

MELLIN'S FOOD.—This food is too well known to require any words of commendation from us. It is extensively used and always gives satisfaction. If you have not tried it, write to Doliber, Goodale & Co., Boston, for a sample bottle and try it. You will be pleased with it.

JOHANN HOFF'S MALT EXTRACT.—We desire to call the attention of our readers to the new advertisement of this preparation to be found next to "Book Reviews" in this issue of THE JOURNAL. It bears the indorsement of the best authorities everywhere. See the advertisement and write to the American agents, the Eisner & Mendelson Co., Philadelphia, for it.

